# Long-Term Follow-Up of Single-Fiber Multiple Low-Intensity Energy Laser Ablation Technique of Benign Thyroid Nodules

**DOI:** 10.3389/fonc.2021.584265

**Published:** 2021-12-07

**Authors:** Mattia Squarcia, Mireia Mora, Gloria Aranda, Enrique Carrero, Daniel Martínez, Ramona Jerez, Ricard Valero, Joan Berenguer, Irene Halperin, Felicia A. Hanzu

**Affiliations:** ^1^ Department of Neuroradiology, Hospital Clínic, Barcelona, Spain; ^2^ Group of Endocrine Disorders, Institut d'Investigacions Biomèdiques August Pi i Sunyer (IDIBAPS), Barcelona, Spain; ^3^ Endocrinology and Nutrition Department, Hospital Clinic, Barcelona, Spain; ^4^ Department of Medicine, Faculty of Medicine, University of Barcelona, Barcelona, Spain; ^5^ Department of Anesthesia and Critical Care Hospital Clinic, Barcelona, Spain; ^6^ Department of Pathology and Anatomy, Hospital Clinic, Barcelona, Spain

**Keywords:** single fiber, unique session, thyroid nodules, long-term response, short-term response

## Abstract

**Aim:**

The short-term and long-term efficacy of different thermal percutaneous ablation techniques remains a topical issue. Our group implemented percutaneous laser ablation (LA), a moving-shot technique to increase efficiency and reduce costs and variability of LA by applying multiple lower-intensity energy illuminations (MLIEI) covering the nodular volume (V) through changes in position of a single laser fiber within the thyroid nodule. The aim of the present study was to evaluate the efficacy of the single-fiber LA-MLIEI during a 5-year follow-up and to identify possible predictors of the final outcome.

**Methods:**

*Prospective study*: Thirty outpatients (23 women and seven men) with benign symptomatic thyroid nodules were assigned to single-fiber LA-MLIEI, between 2012 and 2015. A single LA session was performed under real-time ultrasound (US) guidance using a 1,064-nm continuous-wave laser at 3 W. A 400-µm optical fiber was inserted through a 21-gauge needle, and 3–10 illuminations were performed per nodule, administering between 400 and 850 J/illumination. The total administered energy was calculated on the initial V of the nodule and the estimated ablation area. US evaluation was performed after LA-MLIEI at 1 week and 1, 3, 6, and 12 months and after that annually up to 5 years. Clinical symptoms, laboratory thyroid function during follow-up, and acute and chronic complications of treatment were registered.

**Results:**

On follow-up, 67% (n: 20) were responders to single-fiber LA-MLIEI, while 33% (n: 10) were non-responders. The responder group initiated V reduction (ΔV) at 1 month, with remission of symptoms, and presented a 50% ΔV at 3 months of treatment; the maximum response was achieved at 24 months and remained stable until the end of the study. The non-responder group presented a ΔV of less than 50% at 12 months; though a tendency to >50% ΔV was observed at 24–36 months, there was subsequent regrowth, and 40% of this group required surgery. ΔV was positively correlated with the total administered energy/V (J/V) and inversely with nodule V. No severe adverse effects were observed. Thyroid function remained normal in all patients. Remission of symptoms occurred rapidly after 1 month.

**Conclusions:**

LA with multiple fractional discharges employing a single fiber in a unique session is a safe and inexpensive technique that allows rapid reduction of thyroid nodules, with a stable response up to 5 years, similarly to what has been reported with the conventional LA. Total nodule volume appears as a predictive factor of the reduction.

## Introduction

Thermal ablation of thyroid nodules with laser ablation (LA), radiofrequency ablation (RFA), and very recently microwaves are nowadays increasing minimally invasive percutaneous non-surgical techniques employed in the treatment of symptomatic thyroid nodules that are benign at cytological assessment (1–5). Moreover, they are under evaluation in the local treatment of malignant nodules (6–11).

Initial results from heterogeneous studies comparing RFA and LA showed significantly better results in volume (V) reduction for RFA than LA, but LA included patients with larger nodules, and RFA included patients with a higher cyst component. In the last years, results from both prospective and retrospective multicentric studies showed that both techniques induced a clinically significant 1–5 years of long-lasting V reduction, as well as symptom improvement and preserved thyroid function in treated benign thyroid nodules (12–22). The probability of regrowth needing retreatment was lower after RFA and was associated with a younger age, larger baseline V, and treatment with lower energy delivery (18). Moreover, nodule decrease seemed to initiate earlier for RFA (12, 20).

Both LA and RFA thermal percutaneous techniques are well tolerated and demonstrated to be safe with an extremely low incidence of major local acute or chronic side effects, depending more on the operator experience and the intensity of the applied treatment than on the type of the technique (22–25).

RFA technique consists of moving-shot ablation using a sole probe (5, 12, 14, 16, 18, 20, 26). During LA, the number of fibers employed during each treatment session depends on the nodule V, with mostly one to two illuminations for each fiber (5, 13, 15, 17, 18, 20, 22, 25, 27). Although LA is considered a cost-effective technique in a sole treatment, the use of multiple fibers per session and the eventual need for retreatment increases the costs, generating significant limitations for its use by public healthcare systems.

The aim of the present study was to assess a modified LA technique that intends to improve treatment response, reduce costs, and, therefore, increase efficiency. We employed a single active LA fiber in a unique session treatment covering the nodular V through moving shots and repositioning the optic fiber with multiple lower-intensity energy illuminations (MLIEI). In this work, we report the clinical results, safety, and possible predictors of the outcome of LA-MLIEI during a 5-year follow-up.

## Material and Methods

### Study Design

A prospective one-center non-randomized pilot study was performed in patients with thyroid nodules with benign cytology (single thyroid nodule or a multinodular goiter with a dominant nodule) requiring permanent treatment due to the size and/or compressive symptoms and who either rejected surgery or could not be operated for medical reasons.

### Patients

Patients with symptomatic benign thyroid nodules who rejected surgery or presented a contraindication for surgery due to their comorbidities were admitted between 2012 and 2015 at the hospital outdoor clinic of the Endocrinology Department of Hospital Clinic Barcelona. All nodules showed benign ultrasound (US) characteristics. Fine-needle aspiration (FNA) cytology was performed twice, and Bethesda 2 cytology was required in all LA-treated nodules. The study was approved by the Hospital’s Ethics Committee, and written informed consent was obtained from all participants.

*Inclusion criteria* comprised patients with benign symptomatic solid nodules superior to 25 mm in at least one diameter with local compressive symptoms due to the anatomical position regarding esophagus or trachea or cosmetic symptoms; benign cytological findings (at least two, one in the last 12 months); normal free thyroxine (T4); and normal or low thyrotropin [thyroid-stimulating hormone (TSH)].

*Exclusion criteria* were a history of previous external radiotherapy or radioiodine exposure, multinodular goiter without a dominant nodule, overt hyperthyroidism, V, and diameter below inclusion criteria.

### Initial and Follow-Up Evaluations

Clinical evaluation and US assessment of all patients were performed before LA at 1 week and 1, 3, 6, and 12 months after the procedure, and afterward annually. In addition, laboratory follow-up was performed at 1, 6, and 12 months and annually after that.

Local symptoms were evaluated through a questionnaire assessing the presence/absence of one or more of the following symptoms: neck constriction, cervical tenderness, dysphagia, dyspnea, and dysphonia. The clinical assessment of the signs of the nodular goiter was performed by visual inspection (presence of a cervical lump visible at a distance of 1 m from the patient).

Thyroid US was performed using a commercially available US scanner (Siemens Acuson S2000) with a 7.5- to 13.0-MHz linear transducer. The nodule volumes were calculated by the ellipsoid formula by three experienced sonographers in the center at all examination time points.

Laboratory tests included the following: TSH, serum-free thyroxine (FT4), triiodothyronine (T3), and anti-thyroid peroxidase antibodies (anti-TPOAb). Coagulation was evaluated before the procedure. All laboratory tests were performed following standard assays in the central core laboratory of the center.

### Treatment: Ablation Technique

A continuous-wave multidiode surgical laser (Intermedic, 30 W) operating at 1.064 mm at an output power of 3 W was used for the procedures.

According to the protocols and specific guidelines, anticoagulant therapy was switched to heparin, and antiplatelet therapy was discontinued before the procedure.

The procedure was performed under local anesthesia (2% lidocaine at the point of the puncture) and with conscious sedation with intravenous propofol, fentanyl bolus, and ketamine if required.

**Figure 1:**
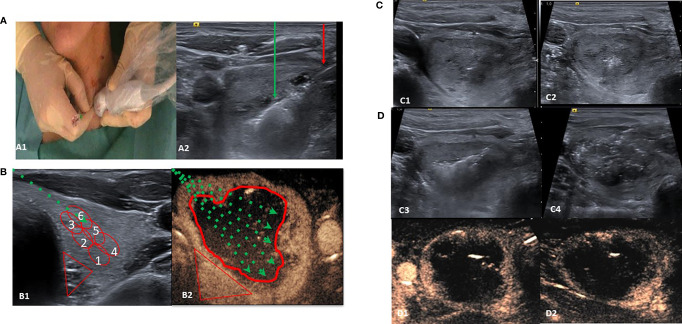
Single-fiber laser ablation multiple lower-intensity energy illuminations (LA-MLIEI) technique. **(A, A1)** Positions of the operator and patient. The patient is placed in a supine position with mild neck extension, and the operator stands close to the patient’s head. The operator’s left hand holds the ultrasound (US) probe and the right hand the electrode. **(A2)** US image of the LA-MLIEI with trans-isthmic approach. This transverse US image shows the needle shaft (red arrow), in the thyroid nodule, and the hyperechoic area (green arrow) from tissue heating and vaporization during illuminations. **(B, B1)** B-mode and drawing of the LA-MLIEI technique. The optic fiber (dotted green arrow) is inserted through the isthmus to visualize the entire length. Illuminations are sequentially performed from the deepest portion of the nodule (red circles Nr. 1 and 4) to the superficial area (red circles Nr. 3 and 6) by pulling back the tip of the optic fiber. **(B2)** Contrast-enhanced US imaging and drawing of the LA-MLIEI technique. The procedure is repeated with different optic fiber angulations (dotted green arrows) to cover all the volume (V) of the target nodule. Illuminations are performed with lower energies in the proximities of the peripheral danger triangle (in red) and higher energies in the central area. **(C)** Sequential US images of the LA-MLIEI technique. **(C1)** Transverse US image showing the tip of the optic fiber. **(C2)** Hyperechoic area in the thyroid nodule appearing during optic fiber firing. **(C3)** Overlapping areas of gas formation during optic fiber moving shot. **(C4)** At the end of the procedure, the complete volume of the nodule appears diffusely hypoechogenic with multiple hyperechogenic gas bubbles dispersed in the treated area. **(D)** Postprocedural axial **(D1)** and sagital **(D2)** contrast-enhanced US images showing complete ablation of the target nodule with the presence of enhancing tissue in the location of the danger triangle, indicating preserved safety margins.

LA-MLIEI treatment was performed by one operator with the patient lying in a supine position with the neck in hyperextension by introducing one guiding 21-gauge needle from the isthmus to the targeted nodule (**Figure 1A1**). Under US guidance, the entire length of the needle was visualized in a transverse US view, and the tip of the needle was initially positioned in the most inferior part of the nodule. A 400-µm optic fiber was then inserted through the needle until the distal active 5 to 7 mm of the fiber was in direct contact with the thyroid tissue (**Figure 1A2**).

LA-MLIEI consists of multiple sequential overlapping low and intermediate energy laser illuminations (between 400 and 850 J/illumination) using a single active optic fiber in an RFA moving shot-like technique. At the end of each illumination, the active tip of the optic fiber was pulled back, and a new illumination was performed in the part of the nodule adjacent to the previously treated area, thus permitting the overlap of the ablated regions. The total number of intranodular repositions of the fiber’s active tip, subsequent illuminations, and administered energy were calculated according to the initial V and the estimated ablation area.

Usually, the procedure began with the trans-isthmic approach at the most inferior part of the nodule, and subsequently, the tip of the needle was withdrawn medially and superiorly for about 8–10 mm with the moving-shot technique. After the ablation of the inferior third of the nodule was completed, the tip of the needle was repositioned at the middle and the superior third of the nodule. Then multiple overlapping illuminations were performed with the moving-shot technique in these locations (**Figures 1B1, B2**).

The single-fiber LA-MLIEI technique allows spreading laser energy in the complete V of the nodule, thus permitting the use of reduced energy illuminations. The peripheral 10 mm of the nodule or the regions of the nodule close to the trachea and the danger triangle were treated by low energy illumination (up to 400 J) to ensure a safe procedure, while the central part of the nodule was treated with higher energy illuminations (between 600 and 850 J) (**Figures 1C1–C4**). To assess the ablated area, a B-mode US, color Doppler, and contrast-enhanced US evaluation (28) were performed immediately after the procedure (**Figures 1D1, D2**).

Methylprednisolone (0.5 mg/kg/*ev*) was administered together with 40 mg pantoprazole *ev* at the beginning of the procedure to prevent airway obstruction by edema. If requested, analgesia was complemented with 1 g of paracetamol. After the procedure, oral corticosteroids were given in a short downward schedule for 5 days (e.g., Urbason^®^ 32 mg/day × 1 day; 16 mg/day × 1 day; and 8 mg/day × 2 days, 4 mg for 1 day) and optional analgesia (paracetamol 1 g/8 h and dexketoprofen 25 mg/8 h vo) if pain persisted.

Tolerability, major/minor complications, and acute/chronic side effects of the treatment were evaluated and registered following previously established criteria. Symptoms were evaluated using symptom questionnaires, and pain intensity was rated by a numeric scale (categorized as mild (<4 of 10), moderate (4–7 of 10), or severe (>7 of 10) (15). The need for subsequent home analgesic treatment or outpatient clinic checkup due to local pain was also registered.

### Statistical Analysis

Continuous variables were expressed as mean ± SD, categorical variables were displayed as frequencies, and the appropriate parametric or non-parametric test was used to assess the significance of the differences between subgroups. All data are expressed as mean ± SD unless otherwise specified. Timing differences within subjects were analyzed through repeated-measures ANOVA or Kruskal–Wallis test for normally or not normally distributed variables, respectively. The correlation between percentage reduction of the nodular V and energy per volume supplies was assessed using Pearson’s method. All the tests were two-sided, and statistical significance was set at p < 0.05. Statistical analysis was performed using the SPSS 20 software package (SPSS, Inc.).

## Results

### Patients and Treatment Characteristics

Thirty patients, 23 women and seven men, fulfilling the inclusion criteria were included in the study. The patients’ clinical, demographic, and laboratory findings are reported in **Table 1**; 36% presented nodular goiter with a single dominant nodule, while 72% presented a unique thyroid nodule. Ablated nodules were localized in the left thyroid lobe in 34% of the patients, in the right thyroid lobe in 25% of the cases, in the isthmus in 28% of the cases, and at the junction of the isthmus left thyroid lobe in 15%. In addition, 6.6% of the patients presented subclinical hyperthyroidism while the rest were euthyroid, and 43.3% presented low (<100 mUI/L) positive TPOAb levels. During follow-up, thyroid function remained normal in all patients. The three patients with subclinical hyperthyroidism normalized thyroid function 6 months after the LA session, without further changes during follow-up. No relationship between thyroid antibodies and treatment or treatment response was observed.

**Table 1:** Clinical, demographic, and laboratory features of all patients at baseline and overall treatment characteristics and side effects.

Variable	
Sex, male/female (n)	7/23
Age (years)	62.3 ± 15.6
BMI (kg/m^2^)	28.2 ± 5.9
Uninodular/multinodular Goiter (%)	72/36
Nodule type (solid/microcystic)	21/7
Thyroid nodule volume (ml)	18.9 ± 21.2
TSH (mU/ml)^a^	2.8 ± 2.3
FT4 (ng/dl)^b^	1.1 ± 0.1
FT3 (ng/dl)^c^	1.1 ± 0.2
TPOAb (mUI/ml) (n/%)^d^	13/43.3
Treatment duration (min)	45 ± 6
Total administered energy (J)	2,954.7 ± 1,689.4
Energy/V (J/ml)	231.9 ± 179.0
Number of illuminations	5.1 ± 7.7
Intraprocedural events (n/%)	1/3.3
Side effects (n/%)	3/9.9

Values are expressed as mean ± SD unless otherwise specified.

BMI, body mass index; TSH, thyrotropin; FT4, free T4; FT3, free T3; TPOAb, anti-thyroid peroxidase antibodies; V, volume; n, number.

a Normal range TSH 0.4–4.0 mUI/L.

b Normal range FT4: 0.8–2.0 ng/dl.

c Normal range FT3: 0.7–1.9 ng/ml.

d TPOAb < 60 mUI/ml.

The nodules’ largest diameter ranged from 26 to 92 mm (median 38.4 mm), and the median volume was 18.9 ± 21.2 ml. Minimal symptomatic diameters were registered for isthmic nodules.

The mean procedure time for LA-MLIEI was of 45 ± 6 min. Between one and five insertions of the unique fiber and between 3 and 10 illuminations were performed for each nodule, releasing between 400 and 850 J/illumination.

### Tolerability and Side Effects

One patient (3.3%) presented stridor during the procedure that remitted with lidocaine administration. Intra- and periprocedural pain was low to moderate in 93% (<7), while in 7%, it was severe. Of the patients, 3% needed complementary analgesia in the first 3–7 days after the treatment.

Of the patients, 9.8% (n = 3) presented an anterior nodular rupture at 6–7 weeks after treatment that remitted with oral anti-inflammatory therapy and prophylactic antibiotic therapy. No other complications regarding treatment or medical examination were registered along with follow-up (**Table 1**).

### Change of Thyroid Nodule Volume During Follow-Up

**Figure 2:**
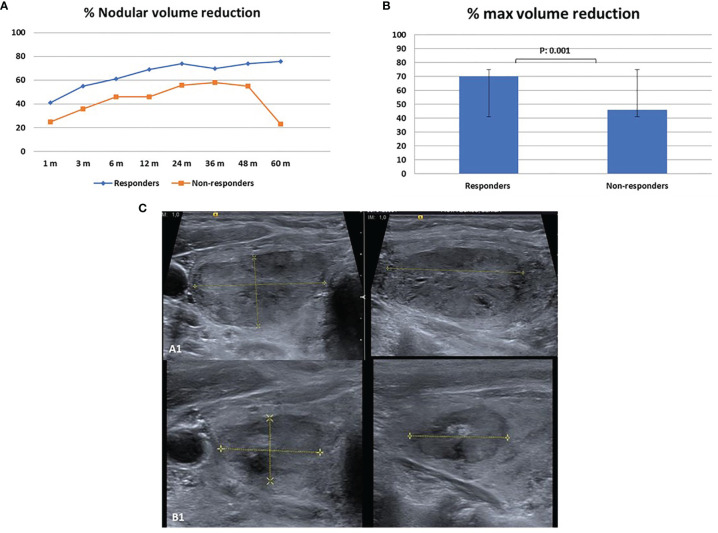
Nodule reduction during follow-up after single-fiber laser ablation multiple lower-intensity energy illumination (LA-MLIEI) technique. **(A)** Percent (%) of nodule volume (V) reduction in the responder and non-responder groups during the 60 months’ follow-up. Values are expressed as mean of % V reduction ± SD. **(B)** Maximum percent (%) of nodule V reduction in the responder and non-responder groups during the 60 months’ follow-up. Values are expressed as mean of % V reduction ± SD. **(C)** Axial and sagittal US images of the target nodule obtained before the treatment and 12 months after the treatment showing a significant volume (V) decrease.

The overall mean V reduction for LA-MLIEI was 35% at 1 month after the procedure, increasing up to 63% at 12 months, and remained 60% at 60 months. Remission of compressive symptoms occurred rapidly after 1 month in 75% of the patients and esthetic symptoms after 3–6 months in 80% of the cases. Four patients (13.3%) were referred to surgery due to persistent symptoms or regrowth during follow-up. One patient was operated because his/her regrowth presented a malignant follicular neoplasia (**Figure 2**).

Two distinct groups of patients were observed according to ΔV reduction in the first 12 months after LA-MLIEI: responders 70% (n: 20) and non-responders 33% (n: 10).

**Table 2** summarizes the clinical and demographic features of responders and non-responders. In the responder group, nodules tended to be more solid and smaller than for non-responders. Furthermore, an increased total energy delivery per nodular V (J/V) during the LA was also observed for the responder group.

**Table 2:** Clinical and treatment characteristics in responders and non-responders to the single-fiber laser ablation multiple lower-intensity energy illuminations (LA-MLIEI) treatment.

Clinical features	Responders	Non-responders	p-Value
Sex, female/male (n)	16/4 (20)	7/3 (10)	ns
Age (years)	60.5 ± 15.1	65.9 ± 16.8	ns
Nodule type (solid/microcystic)	16/4	5/5	0.05
Thyroid nodule volume (ml)	14.2 ± 8.6	30.4 ± 31.6	0.03
Total administered energy (J)	2,816.1 ± 1,840.9	3,232.0 ± 1,383.3	ns
Energy/V (J/ml)	268.3 ± 203.8	159.3 ± 82.3	ns
Number of illuminations	5.5 ± 2.6	6.2 ± 3.8	ns
Intraprocedural events (n/%)	1/3.3	–	ns
Side effects (n/%)	2/6.6	1/3.3	ns

Values are expressed as mean ± SD unless otherwise specified. Statistical significance was set at p < 0.05.

V, volume; n, number.

**Table 3** shows the nodular volume progression in responders and non-responders to LA during 5 years of follow-up. The difference between both groups was statistically significant at every point of evaluation.

**Table 3:** Progression in volume (V) decrease after single-fiber laser ablation multiple lower-intensity energy illuminations (LA-MLIEI) treatment in responders and non-responders during follow-up.

	V 0m (ml)	V 1m (ml)	V 3m (ml)	V 6m (ml)	V 12m (ml)	V 24m (ml)	V 36m (ml)	V 48m (ml)	V 60m (ml)
**LA Responder (n: 20)**	14.2 ± 8.7	9.4 ± 7.5	7.7 ± 6.3	6.6 ± 5.0	5.0 ± 2.7	4.4 ± 2.6	3.7 ± 1.3	3.9 ± 2.0	4.1 ± 2.1
**LA Non-responders (n: 10)**	30.4 ± 31.6	22.3 ± 21.9	20.5 ± 20.3	21.2 ± 26.1	19.8 ± 21.0	18.8 ± 22.8	16.3 ± 4.6	16.7 ± 19.0	17.2 ± 13.6
**p-Value**	0.03	0.02	0.01	0.02	<0.01	0.04	0.03	<0.01	<0.01

Values are expressed as mean ± SD unless otherwise specified.

V, volume; m, months; LA, laser ablation.

The responder group initiated V decrease at 1 month, with remission of symptoms, and presented a 50% V reduction at 3 months of treatment, with a maximum response (74% of V reduction) at 24 months, which remained stable until the end of the follow-up period (**Figure 2**).

The non-responder group presented a V reduction of less than 50% at 12 months, with a later tendency to >50% V decrease at 24–36 months, and subsequent regrowth. Surgical treatment was required by four patients (40%) of this group (**Figure 2**).

### Probable Predictors of Laser Ablation Treatment

Several parameters were analyzed to define probable predictors of nodule reduction: US features (type of nodule), nodular volume, the coexistence of thyroid autoimmunity, and the energy delivered per ml of nodule tissue. Higher energy supplied by thyroid nodule volume (J/V) was correlated with a lower thyroid nodule volume during follow-up (r: −0.477, p: 0.039). Higher total administered energy/V (J/V) and nodule V ≤ 20 ml (p: 0.034) were correlated with a greater ΔV. In other words, the greater the energy applied per thyroid volume, the greater the decrease in the nodule V.

## Discussion

In the present study, we report the short- and long-term results of the single-fiber LA-MLIEI approach to optimize efficiency and reduce conventional LA costs.

Overall, US-guided thermal ablation of thyroid nodules with LA is a safe minimally invasive percutaneous non-surgical technique employed in treating symptomatic benign thyroid nodules and for the local treatment of malignant ones. Various reports regarding the long-term efficacy have been recently published (1–22). What is more, very recently, long-term follow-up compared with RFA showed that both LA and RFA results are clinically similar in volume reduction and sparing thyroid function of benign thyroid nodules with minimal side effects (18, 20, 29).

Conventional LA technique has been implemented using either a variable number of fibers according to the V of the thyroid nodule in one unique treatment or just one in several sessions of LA. The LA technique was improved by the “pull-back technique”, in which one or more laser fibers are subsequently retracted (29). However, generally more than one fiber is required for ablation of each nodule (5, 18, 20, 29–34). Very recently, there has been a report on an intent to standardize the LA technique with a single fiber in a few patients with a 12-month follow-up, with promising results (35).

In contrast, the development of unipolar RFA probes for thyroid nodule ablation and the trans-isthmic moving-shot approach have enabled an outstanding improvement concerning the fixed-electrode RFA technique so that just one probe is required for the ablation of larger nodules (12, 14, 26, 36). As thyroid nodules are often ellipsoidal or exophytic, they are difficult to ablate, and retreatments are sometimes needed to achieve volume reduction or symptomatic remission in LA and RFA (21, 26).

In this work, by applying a standardized approach, we observed a rapid and stable remission of symptoms and a long-term persistent volume reduction of benign thyroid nodules using the LA-MLIEI modified single active LA fiber technique in a single treatment session. The LA-MLIEI technique allows spreading laser energy in the complete V of the nodule, thus permitting reduced energy for each illumination as compared with other reported single- or double-fiber LA techniques (30, 35, 37). We used the LA fiber in a similar trans-isthmic approach, like an RFA probe covering the nodular V through multiple overlapping lower-intensity energy illuminations (MLIEI), repositioning the optic fiber inside the nodule by moving shot. The total number of intranodular moving shot and repositioning of the active tip of the fiber, subsequent illuminations, and administered energy were calculated according to the initial V and the estimated ablation area. At the end of each illumination, the active tip of the optic fiber was relocated, and a new illumination was performed in the part of the nodule adjacent to the previously treated area, thus permitting the overlap of the ablated regions.

During LA-MLIEI, we observed no increase in intraprocedural side effects regarding conventional LA and RFA (23–25, 30, 37).

Interestingly, and probably due to the repeated movements with the fiber through the same initial insert point, the only subacute complication registered was an anterior nodule rupture, a complication that has been described for the RFA technique (25, 38).

Still, nodule rupture did not prevent V reduction, and surgery was not required. The use of conscious sedation can be considered biased for the intraprocedural perceived pain but has allowed us to apply the LA-MLIEI technique following our center’s requirements for minimally invasive procedures. The laryngeal nerves were not affected or there was no dysphonia observed despite the trans-isthmic approach, as the danger triangle was avoided, and a safe distance was carefully kept.

Overall, the volume reduction observed through LA-MLIEI was approximately 50%, in line with the results generally reported for long-time follow-up of LA ablated nodules (15, 18, 22, 29–31, 39). Similar to the RFA technique (12, 16, 20, 30, 36), clinical and US significant reduction started as early as 1 month after treatment. As a result, symptoms improved in more than 75% of the patients. Furthermore, stability of the V reduction was maintained between 6 and 48 months of follow-up, when late regrowth was observed. Treatment did not affect the baseline autoimmunity thyroid reactivity and did not significantly change thyroid function except for the increase of TSH level to the low normal range in the two patients with subclinical hyperthyroidism.

By analyzing the distribution of V decrease and searching for factors predicting V reduction, we observed, just like a recently reported long-term study from conventional LA technique did (39), a dual pattern among treated patients.

The responder group presented a decrease in V starting 1 month after treatment achieving up to 76% of V reduction, which was stable till the end of the follow-up period. The non-responder group showed a slower and inconstant reduction in volume with a significant difference at the 12 months’ follow-up. Moreover, along with the follow-up, the non-responders group showed a tendency for regrowth starting at 36 months, so that more than one-third of this group was finally referred to surgery. One of the four patients who underwent surgery had a final diagnosis of follicular encapsulated thyroid cancer.

Unlike in some previous reports (40), a tendency to produce more solid nodules in the responder group was observed as compared with microcystic nodules in the non-responder group.

Non-responder nodules were larger and received lower energy applied per thyroid volume. These results strongly indicate that probably an increase of at least 30% of the total administered energy for large nodules could improve the LA-MLIEI technique results. This could be achieved either by increasing the level of energy/illuminations in some of the illuminations during the same treatment session or by increasing the number of illuminations by reducing the distance between applications to less than 10 mm.

The LA-MLIEI single-fiber technique permits a flexible nodule ablation, increasing the possibility of approaching the nodule by the operator. Furthermore, in comparison with the multifiber procedure, the time of ablation of the LA-MLIEI single-fiber procedure is longer (50 min in comparison with 30 min), something utterly compatible with ambulatory treatment, while the expenses due to the costs of the optical fiber are significantly lower (300 € in comparison with 600 € or 900 € for multiple fibers).

The relatively small number of patients and the absence of a comparative group are to be considered as the limitations of the present study. However, the strength of this study is its extensive follow-up. Multicenter studies employing the single-fiber LA-MLIEI technique are expected for large-scale use.

In conclusion, LA-MLIEI allows rapid reduction of thyroid nodules and is a safe, effective, and well-tolerated procedure in the treatment of benign thyroid nodules with similar results in V reduction and symptom remission to those reported with conventional LA at 1, 6, and 12 months and up to 5 years of follow-up. Moreover, LA-MLIEI is a cost-effective and easily reproducible procedure since only one active fiber is used in each treatment. Therefore, it should be considered for the treatment of large V nodule candidates who need retreatment.

## Data Availability Statement

The raw data supporting the conclusions of this article will be made available by the authors, without undue reservation.

## Ethics Statement

The studies involving human participants were reviewed and approved by Comite Etico Hospital Clinic Barcelona. The patients/participants provided their written informed consent to participate in this study.

## Author Contributions

MS and FH: primary investigators, involved in study planning, data collection, data analysis and interpretation, and manuscript writing. MM, GA, EC, RJ, RV, and DM: involved in study planning, data collection, data analysis and interpretation, and proofreading of the manuscript. IH and JB: involved in study planning, data collection, data analysis and interpretation, manuscript writing, and proofreading of the manuscript. All authors contributed to the article and approved the submitted version.

## Funding

The project was funded by the Institut d’Investigacions Biomèdiques August Pi i Sunyer (IDIBAPS) by the Instituto de Salud Carlos III (ISCIII) PROMIS II12/00003 grant and from own research funds of the group.

## Conflict of Interest

The authors declare that the research was conducted in the absence of any commercial or financial relationships that could be construed as a potential conflict of interest.

## Publisher’s Note

All claims expressed in this article are solely those of the authors and do not necessarily represent those of their affiliated organizations, or those of the publisher, the editors and the reviewers. Any product that may be evaluated in this article, or claim that may be made by its manufacturer, is not guaranteed or endorsed by the publisher.
